# Green HEMS: how to make it happen

**DOI:** 10.1186/s13049-023-01087-9

**Published:** 2023-05-04

**Authors:** E. ter Avest, M. Kratz, T. Dill, M. Palmer

**Affiliations:** 1 Air Ambulance Kent, Surrey and Sussex, Redhill Airfield, Redhill, Surrey, RH1 5YP UK; 2 University Medical Center Groningen, University of Groningen, Groningen, Netherlands; 3 Emergency Medical Services, Centre for Prehospital Emergency Care, Department of Emergency, Anaesthesia and Pain Medicine, Tampere University, FinnHEMS 30 & 40, Tampere, Finland; 4 Department of Anaesthesiology and Pain Medicine, University Hospital Bern, Inselspital, Bern, Switzerland; 5 Emergency Medical Transfer Retrieval Service- Wales Air Ambulance, Ty Elusen, Ffordd Angel, Llanelli Gate, Dafen, Llanelli UK

Since the industrial revolution the growth of carbon dioxide (CO_2_)-emissions worldwide have been unmitigated. During COVID-19 pandemic, a slight reduction in human-driven greenhouse gas emissions was observed in 2020, yet in 2022 they were the highest ever recorded [1]. Overwhelming evidence directly links the increase in greenhouse gas emissions to the currently observed, unprecedented global temperature increase [2, 3], resulting in the melting of the icecaps, sea-level rises, and extreme weather events making climate change the biggest global health threat of the 21st century [4].

The healthcare industry’s impact on greenhouse gas emissions is significant, accounting for 10% of all US emissions each year [5]. In 2020 the World Health Organization published a Guidance for Climate Resilient and Environmentally Sustainable Health Care Facilities. Recently numerous initiatives have been deployed to reduce the carbon footprint of the healthcare industry, for example by in-hospital colleagues from Emergency departments [6, 7] and operating rooms [8].

Attention for the environmental impact of Helicopter Emergency Medical Services (HEMS) has been limited so far, although HEMS’ impact on CO_2_-emissions is substantial. Helicopters are responsible for a significant part of the carbon footprint in HEMS and burning of fossil fuel (mainly JetA1) is responsible for 95% of the helicopter related emissions [9]. For every kg JetA1 fuel burned, on average 3.16 kg of CO_2_ is released into the atmosphere [10]. Commonly used HEMS aircraft in Europe, the Eurocopter 135P2 or the AugustaWestland 169, use around 270 kg of JetA1 fuel per hour on average. During an average mission with a flight time of 50 min (20 min approaching the patient, 20 min transporting the patient to hospital, and 10 min return flight to base [11]), 0.72 tons of CO_2_ are emitted into the atmosphere. This, to provide a perspective, equals CO_2_-emissions of a 6000 km car journey from Tromso, Norway to Marrakesh, Morocco [10]. Other aviation related environmental aspects warranting consideration include helicopter manufacturing, maintenance, and crew-training.

As the aviation- related carbon footprint in HEMS is overt and easily quantifiable it might seem tempting to discard non-aviation related CO_2_-emissions as trivial. However, looking at examples from elsewhere in health care it is likely there is additional and significant non-aviation related carbon footprint to address, and concurrently with helicopter industry, HEMS should set out policies to reduce CO_2_-emissions identifying both aviation and non-aviation (“medical”) related CO_2_-emission sources of our operations involving leadership commitment and organisational, cultural and systemic changes [9].

Introducing a “green team” and adopting the simple ‘6R’ attitude as described below can be a start for organisations and trusts to evaluate environmental sustainability within their services.

- „Refuse“: Unnecessary flight- and car movements (stand-downs) should be minimised without compromising patient safety. Although a certain degree of over-triage is inevitable in HEMS, a streamlined dispatch process may contribute to a lower number of stand-downs. Careful consideration of dispatch criteria and the use of tools, such as video-assisted dispatch, can help optimise the use of available resources [12].
- “Reduce”: First, when HEMS services look at purchasing or leasing an aircraft, environmental impact data [9] should be considered alongside performance data. Options to deploy sustainable aviation fuel compatible with existing aircraft and fuelling infrastructure should be explored with the manufacturer and the local airfields. Second, consideration should be given to alternative ways of transport: In high built-up areas with sparse landing zones, helicopters may not be the fastest way to reach or transport the patient. Establishing a clear car-footprint tailored to the local situation may help decide when helicopter dispatch can benefit the patient, and when not. Considering that a significant amount of HEMS missions is carried out using rapid response vehicles during no-fly-conditions, the use of electric response cars should be considered. Third, bulk-ordering medical material (if expiry dates allow) can minimise packaging and reduce the environmental impact of transport. Further, general environmentally sustainable practices should be introduced around base including the provision of reusable kitchenware, replacing bottled water and beverages with fountains or tap water, providing local-, seasonal- and plant-based food (if meals are provided on base) and conserving energy when and where possible (room temperature reductions, turning off un-used lights, screens, etc.). In designing new bases renewable energy, energy efficiency, resource and water conservation, and sustainable building materials should be considered as well as the use of space to promote and enable environmentally friendly practices (e.g. possibility to stay on base between shifts to help to reduce commute related emissions).
- “Reuse”: Limiting the use of disposables and investing in reusable medical and office equipment will reduce waste and possibly costs [13]. Examples include re-usable (video-) laryngoscopes and blades, head blocks, and blankets. Further, consideration should be given not to automatically discard of drugs exposed to conditions outside of manufacturer‘s recommended range. Real-life stability testing has shown that drug exchange intervals can be extended safely beyond the manufacturer’s recommendations on many occasions [14].
- „Repurpose“: Redistributing blood products and rarely used drugs and devices before they expire to other facilities with a higher turnover may help reduce wastage of these (often also expensive and sometimes scarce) products.
- „Recycle“: Returning waste to base and recycling equipment and drugs’ packaging should be the norm. As HEMS providers, we rely on high quality protective gear and workwear that need to comply with safety regulations. The clothing industry is one of the largest polluters globally, therefore repairing and maintaining workwear and gear properly are essential. However, when choosing a new set of gear, equipment or office supply we should consider purchasing from companies that invest in eco-friendly development and production.
- „Rethink“: Taking strategic perspectives that embrace wider range of levers – thinking outside the box - we can achieve solutions and innovations not yet obvious to us. An example of this are electric vertical take-off and landing (eVTOL) aircraft. Although conventional helicopters will likely remain essential to perform HEMS, and search and rescue missions in the near future, there may be a place for eVTOL, especially in rural area’s, where it may serve as a first response, to support ground crews or to allocate resources more aptly for example by providing live video footage from the scene [15, 16].

With this commentary we offer examples of how HEMS practice can become more sustainable and call on the HEMS community to engage in discussion and actively look at feasible options adapted to their local situation to reduce our cumulative carbon footprint. We feel that as a community, we all have a responsibility in addition to our patient care, to address the global health threat we are facing, and that it is in line with our key mission of saving lives.

### Electronic supplementary material

Below is the link to the electronic supplementary material.

Supplementary Material 1

## Data Availability

Not applicable.
