# Supplementary material for: Green HEMS: how to make it happen
Source: Scand J Trauma Resusc Emerg Med. 2023 May 4;31:23. doi: 10.1186/s13049-023-01087-9 (PMC10158689; doi:10.1186/s13049-023-01087-9)
Supplement: Supplementary file 1 — Supplementary Material 1 [file 13049_2023_1087_MOESM1_ESM.docx]

Prof. Hans Morten Lossius Editor-in-chief

Scandinavian Journal of Trauma Resuscitation and Emergency Medicine

| Date | March 28^th^ 2023 |
| --- | --- |
| JOur reference | Manuscript submission  Manuscript number: TBA |

Dear Editor,

**“Green HEMS: How to make it happen.”**

**.**

We have pleasure in submitting this manuscript for publication as a “commentary” in your journal.

Since the industrial revolution, growth of carbon emissions worldwide has been unmitigated. Overwhelming evidence directly links the growth in emissions to the currently observed and unprecedented global temperature increase. This makes climate change the biggest global health threat of the 21^st^ century.

The healthcare industry’s impact on greenhouse gas emissions is significant. Attention for the environmental impact of the workplace in Helicopter Emergency Services (HEMS) has been limited so far, although HEMS’ impact on CO_2_-emissions is substantial. Although helicopter transport is an inevitable part of HEMS, there are many ways CO_2-_emissions can potentially be reduced.

With this commentary we offer examples of how HEMS practice can become more sustainable and call on the HEMS community to engage in discussion and actively look at feasible options adapted to their local situation to reduce our cumulative carbon footprint.

We think our thoughts will be of value to your readers, and will contribute to the scope of your journal.

All authors have made a substantial contribution to the conception of the study, to acquisition of the data and/or analysis and interpretation of the data, and to drafting the manuscript or revising it critically for important intellectual content. All authors gave final approval of the version to be submitted. None of the data presented in this manuscript has been published before, and the manuscript is not under consideration elsewhere.

We look forward to hearing from you,

With best regards,

Dr. E. ter Avest, on behalf of the co-authors
